# Carbon Footprint: The Case of Four Chicken Meat Products Sold on the Spanish Market

**DOI:** 10.3390/foods11223712

**Published:** 2022-11-18

**Authors:** Harrison Tetteh, Alba Bala, Pere Fullana-i-Palmer, Mercè Balcells, María Margallo, Rubén Aldaco, Rita Puig

**Affiliations:** 1 Department of Computer Science and Industrial Engineering, University of Lleida (UdL), Pla de la Massa, 8, 08700 Igualada, Spain; 2 UNESCO Chair in Life Cycle and Climate Change ESCI-UPF, Pg. Pujades 1, 08003 Barcelona, Spain; 3 Department of Chemistry, University of Lleida (UdL), Rovira Roure 191, 25198 Lleida, Spain; 4 Department of Chemical and Biomolecular Engineering, University of Cantabria, Av. de Los Castros s/n, 39005 Santander, Spain

**Keywords:** life cycle assessment, greenhouse gas emissions, poultry meat, whole carcass and meat cuts, allocation to meat cuts

## Abstract

Despite its relatively low environmental impact within the livestock sector, the poultry sector still faces its own environmental challenges that need to be addressed. The present paper uses life cycle assessment to quantify greenhouse gas emissions, from cradle to slaughterhouse gate, of four chicken meat products: whole carcass, wings, breast fillets, and leg quarters. The main contribution of the present study is that it provides a detailed analysis of different chicken meat cuts, testing mass and economic allocation choices and showing that economic allocation better reflects the causality of the cutting process. We recommend that a distinction should be made between whole carcass and meat cuts, as there are significant differences in meat content and climate change results between these two categories. This is not so clear in the literature, nor in the LEAP guideline for the poultry sector. The study was performed by using disaggregated inventory data from Spain, for the first time. Results show that the major contributors to environmental impact are feed production (>70%), electricity use (10.2%), and fossil fuel combustion (8.1%). Packaging did not significantly contribute to the climate change impact of the chicken products evaluated (0.4–3.4% contribution, depending on the type of packaging and product considered).

## 1. Introduction

The European Union (EU) is currently the second largest producer of meat after China, and one of the largest consumers of animal products per capita [1]. Out of 44 million tonnes of meat produced in 2020, pig meat constituted the highest share (52%), followed by poultry meat (31%), beef and veal (16%), and sheep and goat meat (1%) [2]. However, according to the European Commission [3], meat consumption per capita has started to decrease and this is expected to continue into 2030. This downward trend is due to changes in consumer preferences in favour of healthy and sustainable products [1,4]. In contrast to this expected decline in the production and consumption of meat products, poultry meat production and consumption are expected to increase during the same period, because consumers perceive poultry to be a healthier and more sustainable alternative to other meat products [4]. Currently, broiler meat represents 82% of the total poultry meat produced in the EU-27 [5]. Poultry meat (including eggs), with an overall contribution to climate change of 9.8%, has the lowest climate change impact per kg of meat when compared with other popular animal meat products, such as beef (37.0%) and pig meat (10.1%) [1]. There is also evidence that a shift from a relatively high resource-intensive meat, such as beef, to a low resource-intensive meat, such as poultry, could reduce greenhouse gas (GHG) emissions by up to 50% [6]. However, despite the relatively low environmental impact of poultry meat, the poultry sector still has environmental challenges to overcome [7]. Some of the main areas needing improvement according to environmental impact assessments include feed production and transport [8,9], fossil fuel combustion, electricity use, and emissions from manure storage and management [8,10].

Life cycle assessment (LCA) is the most commonly used environmental management tool to assess the environmental impact of the livestock sector [11]. LCA involves quantifying the environmental impact of a product (whether a consumer good or service) across its life cycle [12,13]. Many environmental impacts can be assessed using LCA. However, a product’s global warming potential (GWP), or climate change impact category, has become a great concern for businesses, governments, and the public, because of its environmental and socio-economic effects [14]. Additionally, the livestock sector is an important source of anthropogenic GHG emissions [15]; therefore, studies focusing on understanding the reasons and sources of these emissions are needed to complement global efforts to mitigate climate change [16]. There are international guidelines for evaluating the GHG emissions of products [14,17], but most of these provide general guidance that is not specific to the poultry sector. An exception to this is the Livestock Environmental Assessment and Performance Partnership (LEAP), developed by the Food and Agriculture Organisation [18]. The LEAP provides guidance for assessing GHG emissions and fossil fuel energy use in poultry supply chains. This sector-specific guideline aims to make comparisons of LCA studies in the poultry sector easier, by homogenising methods for assessment. Therefore, the present paper applies the LEAP recommendations, especially in allocating environmental burdens in multi-functional processes in broiler production systems.

There are three common broiler production systems [19]: (1) Standard or conventional systems, where birds are housed in barns with lighting, heating, or cooling and the floors are layered with bedding material; (2) Free-range or extensive systems, where birds are free to roam around the farm; (3) Organic systems, where birds are raised in housing with outdoor access and provided with organic feed and regulated treatments. The present paper only focuses on LCA studies assessing the impacts of the conventional system.

Various chicken meat LCA studies were found in the literature (see Tables 1 and 2). These studies used different approaches (country, scope, functional unit, type of allocation considered, etc.) which makes comparison difficult. Chicken meat LCA studies have been performed at different geographical levels (Tables 1 and 2), including at country [8,10,20], continent [21], and global levels [22]. Some of these provided scientific explanations for variations in the results of similar studies across different parts of the world [8]. These regional variations were due to differences in feed consumption depending on climate, feed composition and origin, nitrous emissions and fossil fuel use in crop production, and differences in electricity emission factors [8]. Therefore, country-specific studies are needed, to serve as standards for future research and provide guidance for stakeholders and policy-makers in the poultry supply chains. Spain is among the major producers of poultry products in Europe, with a 13% share of the total poultry production in 2020 [5]. Despite this, peer-reviewed LCA studies in the Spanish poultry sector are lacking. Currently, there has only been one study on egg production [23], whereas for chicken meat production, to the best of our knowledge, there has only been one conference publication [24]. Thus, the present paper aims to fill this research gap.

In addition, disaggregated inventory data for the different broiler farm stages (breeder, hatchery, and grow-out) has only been presented in two published studies [8,25]. The rest of the papers either only presented data for the grow-out farms or did not present separate inventory data for the various farm stages. Moreover, some studies presented results on whole carcass and/or meat cuts/portions [8,26]. However, no detailed analysis was provided to explain significant differences in the impact results between whole carcass and meat cuts (wings, breasts, and leg quarters). In this regard, the present paper contributes to these gaps in the literature by presenting detailed inventory data for the different farm stages and analysing methodologies to allocate impacts according to whole carcass and different meat cuts.

Thus, the aim of the present paper is to perform detailed analyses of several commercial chicken meat products (whole carcass and chicken meat cuts/portions) to identify the most convenient allocation method to be applied. The study also fills several gaps in the literature, such as evaluating the GHG emissions of chicken meat production in Spain and providing separate disaggregated inventory data for each broiler farm stage (rarely shown in the literature).

## 2. Literature Review of LCA Studies Applied to Chicken Meat Production

In this section, a literature review of chicken meat production LCA studies is presented in two different tables. Table 1 presents peer-reviewed papers related to live chicken production (before slaughterhouse), whereas Table 2 shows chicken meat production (including slaughterhouse). Geographical level, methodologies used, and results obtained, are also shown in the tables. Comparisons of the different approaches used and possible reasons for the differences in results are discussed.

As presented in Table 1, four papers in the literature assessed chicken production from cradle-to-farm gate (not considering the slaughterhouse) and four papers considered only the grow-out farm (excluding the breeder farm and hatchery). From the first group (cradle-to-farm gate), GHG emission results per kg of live chicken was between 1.28–1.39 kg CO_2_ eq. The result of one study from Iran [27] was beyond those margins with 6.83 kg CO_2_ eq./kg live weight. In this case, this high result was not explained, and inventory data was not presented. Another paper [7] did not study the impacts from the slaughterhouse but nevertheless presented the expected results per kg of edible carcass (4.41 kg CO_2_ eq./kg), and thus was not comparable with the others. From the second group (including only grow-out farm), the calculated range was 1.33–2.70 kg CO_2_ eq./kg live-weight. Overall, results were widely varied, due to the heterogenous nature of the reviewed studies.

The factors that can influence results are the scope of the study, allocation method considered, type of feed, climate, and performance indicators for poultry production (such as FCR—feed conversion ratio, days to slaughterhouse, and mortality). Regarding the scope of the study, only two papers [25,28] considered cradle-to-farm gate (including spent hens, fertilised-egg production, and hatchery).

Allocation may be performed when a process results in more than one product. In this case, the burdens of the process need to be shared among all the products obtained. For example, in the breeder farms, one-day-old chicks are fed to produce hens which will, in turn, produce fertilised eggs. Therefore, burdens of the breeder farm need to be allocated to two main products, which are the fertilised eggs and spent hens. The proportion of the burden corresponding to each product can be established according to its relative mass, economic revenue, or others. Some studies use economic allocation, whereas others use other types of allocation (e.g., gross chemical energy content, metabolized energy, etc.) (see Table 1). Manure, another byproduct obtained in the breeder and grow-out farms, was treated as a residual in the present study. Hence, no allocation was made between it and the main products from the farms. The residual status of manure implies that impacts associated with its management on the farm are allocated to the farm itself, whereas its downstream impacts are cut off. Nevertheless, some studies assign credits to the farm for the manure because it can be used as a fertiliser, substituting the production of chemical fertilisers [28].

Finally, it is worth mentioning that though there were only four papers with the same scope (only grow-out farm) and no allocation considered, results between them widely varied because of differences between feed composition and performance indicators of poultry production. Additional comments related to inventory data provided by these four papers are:

Only one [25] included spent-hen meat destined for human consumption and successive breeding generations into the analysis of broiler production.

Only two studies presented primary inventory data from farms [25,29], the rest used secondary inventory data from literature.

**Table 1:** Literature review on LCA studies of live chicken production.

						Allocation	Result
Reference	Country	Scope	Functional Unit (FU)	FCR (kg Feed/kg Weight Gain)	Days to Slaughter/ % Mortality	Breeder Farm	kg CO_2_ eq./kg FU
[28]	United States	Cradle-to-farm gate	1 t live-weight poultry	1.9	48 days NS	1. Gross chemical energy content 2. Manure replacing chemical fertilisers	1.39
[7]	United Kingdom	Cradle-to-farm gate	1000 kg of expected edible carcass	NS	39 days 3.5%	Economic	4.41
[27]	Iran	Cradle-to-farm gate	1 kg live chicken	NS	NS NS	NS	6.83
[25]	United States	Cradle-to-farm gate	1000 kg of live poultry and spent hens	1.94	47 days 4%	Biophysical: metabolised energy	1.28 (in 2010)
[30]	Argentina	Only grow-out farm	1 t live chicken	2.02	49.5 days 6.85%	NA	2.03–2.22
[29]	Brazil	Only grow-out farm	1 kg live chicken	1.89	50 days 3.8%	NA	2.70
[31]	Brazil	Only grow-out farm	1 kg live chicken	1.40–1.82	28–49 days 3–4%	NA	1.33–1.56
[32]	Japan	Only grow-out farm	1 kg live chicken	1.98	52 days NS	NA	1.86

FCR: feed conversion ratio; NS: not stated; NA: no allocation.

In Table 2, as previously mentioned, LCA papers that studied the chicken meat production (bird production and slaughterhouse) were compared. A total of 11 papers assessing the environmental impact of chicken meat production were found. The scope of the studies varied from one another: most of them studied impacts from cradle-to-slaughterhouse gate, two from cradle-to-retail, and the last one from cradle-to-grave (including chicken meat consumption and end of product life). When comparing the eight papers that assessed impacts from cradle-to-slaughterhouse gate (same scope as the present study), the results were largely in the range of 2.46–3.30 kg CO_2_ eq./kg carcass. However, the results of two of the studies were beyond this range: 5.52 kg CO_2_ eq. [33] and 1.99 kg CO_2_ eq./kg carcass [9]. The latter is a study from Brazil, and according to the authors, the relatively low impact was because there was no deforestation impact of feed crop production, which was obtained from the South of Brazil. Nevertheless, this study had two weak points: first, due to lack of data on Brazilian chicken rearing, French data was used instead, and second, the survey data lacked replication. 

**Table 2:** LCA studies of chicken meat production.

				Allocation		Result
Reference	Country	Scope	Functional Unit (FU)	Breeder Farm	Slaughterhouse	kg CO_2_ eq./kg FU
[34]	Finland	Cradle-to-retail gate	1000 kg honey-marinated and sliced broiler fillet at retail store	NS	NS	3.64
[26]	Australia	Cradle-to-retail	1 t of roast chicken	Economic Economic	Economic Economic	3.71
			1 t of breast fillet			9.98
[35]	Reunion Island, France	Cradle-to slaughterhouse gate	1 t packed whole carcass	1. Economic 2. Avoiding allocation for manure by substitution.	1.Economic 2. Avoiding allocation for waste used as fertiliser by substitution	2.48
[9]	France Brazil	Cradle-to-slaughterhouse gate	1 t of cooled and packaged chicken	NS NS	NS NS	3.10 1.99–2.75
[20]	Portugal	Cradle-to-slaughterhouse gate	1.2 kg of broiler chicken meat	NA	No allocation	2.46
[36]	Iran	Cradle-to-slaughterhouse gate	1 t of packed chicken meat	NS	NS	2.9 (summer)–5.3 (winter)
[33]	Italy	Cradle-to-slaughterhouse gate	1 kg chicken carcass	NA	No allocation	5.52
[37]	Tunisia	Cradle-to-slaughterhouse gate	1 kg chicken carcass	NS	NS	3.3
[8]	Australia	Cradle-to-slaughterhouse gate	1 kg of chilled chicken (whole bird)	NA NA	Economic Economic	2.8–3.4
			1 kg of boneless, skinless chicken portions			3.9–4.79
[38]	Mexico	Cradle-to-slaughterhouse gate	1 kg chicken carcass	Mass	Mass	2.79
[10]	Serbia	Cradle-to-grave	1 kg chicken meat	Mass	Mass	3.62

NA: no allocation; NS: not stated.

Additional comments related to the eight papers highlighted in grey in Table 2 are:

Only two papers [8,33] presented data from breeding, hatching, and grow-out farms.

Only one paper [8] presented disaggregated inventory data from the different farms.

The rest of the papers presented only farm performance data [9,33], or aggregated inventory or inadequate data (because they only focused on feed production [37] or on process simulation [38]).

Thus, further LCA studies of chicken meat production allowing for the performance of uncertainty analysis [9] are needed. These studies should provide data representing country-specific performance from several farms and slaughterhouses, and show variability around mean data.

## 3. Materials and Methods

As the aim of this study was to evaluate the GHG emissions in chicken meat production using a life cycle approach, we used the methodologies for life cycle assessment (LCA) and product carbon footprint, as specified in the ISO standards in [12,13,14], respectively. In addition, the poultry-specific LEAP guideline [18] was also used, especially for allocating burdens in multi-functional processes. The next sub-sections follow ISO standards for performing an LCA study.

### 3.1. Goal and Scope

This study was commissioned by a major player in the chicken meat industry in Spain, with an annual production of about twenty-four million birds (24,000,000). The company is vertically integrated with farms across multiple regions and municipalities in Spain. The company’s motivation for the study was to understand the GHG emission hotspots within its supply chain, in order to address their mitigation. Moreover, GHG emission results are the most compared environmental impact assessment results within the poultry meat sector [26]. Therefore, only one impact category, climate change (CC), excluding biogenic carbon [kg CO_2_ eq.], was evaluated, using the ReCiPe 2016 v 1.1 Midpoint (H) method [39,40]. The modelling of the systems studied was done using GaBi ts 10 software [41]. Table 3 shows the main chicken meat products studied. A byproduct (byP6) mostly consisting of bones and cartilages was also evaluated, but it is only presented in Table 4 and the results section.

**Figure 1:**
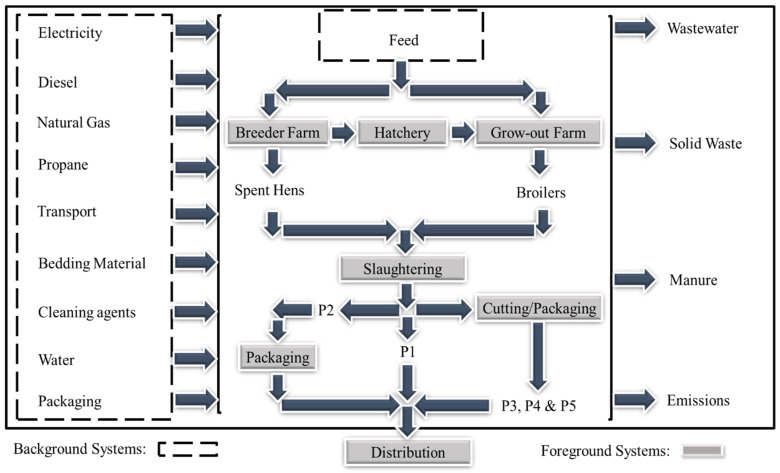
System boundaries for chicken meat production.

**Figure 2:**
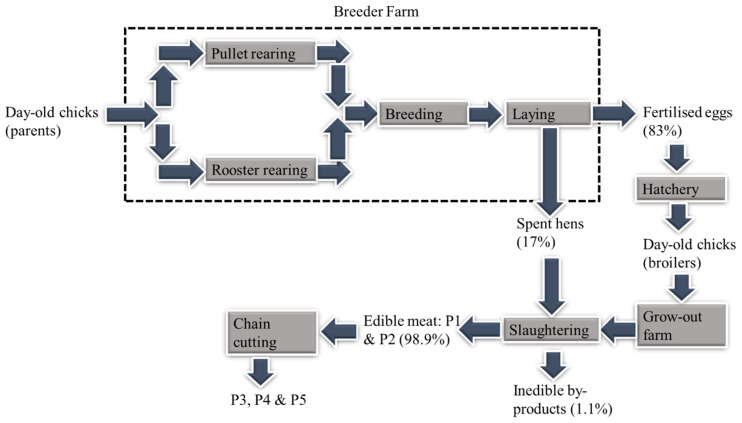
Simplified process flow of chicken meat production with allocation percentages of multi-functional processes related to the edible whole carcass.

A cradle-to-slaughterhouse gate life cycle approach was used for the GHG emissions evaluation. This included all processes and their emissions, from feed production to the exit gate of the slaughterhouse, where products were packaged for distribution. Figure 1 shows the system boundaries of the chicken meat products evaluated. The breeder farm consists of both rearing and breeding barns. In the rearing phase, one-day-old chicks are raised for about 147 days, after which they are sent to the breeding phase. At the breeding phase, both roosters (males) and pullets (hens) are raised together until the hens begin laying. The laying phase lasts for about 278 days. The fertilised (best quality) eggs are transferred to the hatchery for incubation and hatching of the broiler chicks. The broiler chicks are raised in grow-out farms for about 44–45 days before they are slaughtered for meat (see also Figure 2). The functional unit (FU) selected for this study was 1 kg of each chicken meat product evaluated from cradle-to-slaughterhouse exit gate (weight of packaging was not included).

In systems with multi-functional processes (e.g., broiler production), allocation is a common option. Nonetheless, allocation is one of the challenging decisions in an LCA study because of its potential to affect the outcome of the study and make it difficult to compare results across similar studies [18,26]. This is where the LEAP guideline in poultry supply chains becomes very useful. The LEAP guidance on allocation follows the ISO 14044 recommendation, which is to avoid allocation and expand the system if possible [13]. However, in situations where allocation is unavoidable, the guideline provides recommendations on how to allocate burdens in multi-functional processes. In the present study, mass and economic allocation principles were used in the breeder farm and slaughterhouse in accordance with the LEAP guideline [18]. In the breeder farm, mass allocation was used to allocate burdens to spent hens (17%) sent to the slaughterhouse, and fertilised eggs (83%), sent to the hatchery. In the slaughterhouse, economic allocation was used to allocate burdens to the edible meat (98.9%) and inedible byproducts (1.1%) as shown in Figure 2. 

Apart from the edible whole carcass, this study also analysed meat cuts obtained by cutting the whole carcass into different portions (wings, breasts, and leg quarters). For these meat cuts, both mass and economic allocations were used to evaluate the impact of allocation choice on the result. The LEAP guideline does not distinguish between different meat cuts and the edible whole carcass. The guideline recommends that these are treated as equivalent and therefore, no allocation is given to the different meat cuts. However, considering that the company needs to evaluate all its meat products, it was necessary to consider the meat cuts separately. Moreover, previous studies have shown that the meat cuts have higher impacts than the whole carcass [8,26]. Therefore, further analysis on the meat cuts using mass and economic allocations were performed (see Table 4). It is important to note that, after dividing the whole carcass into meat cuts, an edible offal is produced (byP6), consisting of bones, cartilages, and flesh. Part of this may be sold for human consumption and the rest used as animal feed.

Manure is a usual byproduct of livestock production. In poultry production, the LEAP guideline recommends that the classification of manure as a co-product, residual, or waste for allocation purposes should be based on its revenue generation to the farm. When manure is considered a co-product, the farm generates substantial revenue from it, and so environmental burdens are allocated to it just like the other main products. When manure is considered a residual, the farm does not generate any (or little) revenue from it, but it has subsequent use in other systems. Therefore, impacts associated with its on-farm management are allocated to the farm, whereas its downstream impacts are cut off. In instances where manure is considered waste, it generates no revenue to the farm and has no subsequent use in other systems. In this case it carries no burdens, but the impacts associated with activities related to its treatment and disposal are assigned to the farm. In this study, manure generated on the farm was used by vegetable growers as a replacement for synthetic fertiliser, but the poultry farm did not make substantial revenue from it. Therefore, it was treated as a residual, which implies that emissions from its storage, management, and transport to the vegetable farms were allocated to the poultry farm while emissions due to its downstream use were cut off [18].

### 3.2. Inventory

Both primary and secondary inventory data were used for this study. Primary data was collected through questionnaires submitted to various farms within the study scope and was based on production data for the period 2011–2012. There was no significant difference between the production data obtained within the stated period and current production. On the other hand, secondary data was sourced from LCA databases, mostly from a GaBi database [41] and the scientific literature. Capital goods were excluded from this study, based on the LEAP recommendations that if the lifetime of the building and machinery is greater than a year, their impacts can be ignored [18]. Rendering materials (e.g., dead and culled birds) from breeder farms, grow-out farms, and hatcheries were treated as waste [18]. Although rendering companies extract fat and other useful products from this waste, the emissions from the transport and rendering processes were excluded from this study due to lack of data. Impact from vaccines used on the farms was also left out of the calculations, because of lack of data on active ingredients. The transport of these vaccines was also excluded because of its relatively small contribution to the freight transport, less than 1% [18]. Wastewater from the hatchery and slaughterhouse has a high organic load; therefore, it requires treatment before its discharge into the municipal wastewater system [42]. The sewage sludge generated from the wastewater treatment is composted. However, the impact from the composting process was excluded because the process was carried out by an external company, and it was difficult to obtain data. The volume of wastewater generated on the farms was estimated by subtracting the volume consumed by the birds (1.9 L/kg consumed feed) from the total volume of water supplied to the farms. The wastewater treatment was modelled using a GaBi database.

Diesel, propane, natural gas, and biomass were the main fuels burned to supply heat to the farms. The GHG emissions from the combustion of these fuels, except biomass, were modelled using GaBi databases. Carbon dioxide emissions from biomass combustion were excluded because it was assumed that they were balanced by the CO_2_ absorbed during photosynthetic growth of the biomass [43]. Moreover, a previous chicken meat LCA study [20] found that CO_2_ emission from biomass combustion on poultry farms was relatively small. Electricity use on the farms and in the slaughterhouse was modelled using the Spanish medium voltage electricity grid mix (1–60 kV), available in the GaBi database. Freight transport (tonne-kilometre: tkm) included transport of inputs and animals/products within the supply chain evaluated in this study. This was modelled using the freight transport data available in GaBi. Emission factors of methane (CH_4_) and nitrous oxide (N_2_O) from enteric fermentation were obtained from a previous study [44], whereas those from manure storage and management were obtained from the IPCC guidelines [45].

#### 3.2.1. Feed Production

Secondary data was used to assess the impact of feed production on the chicken meat GHG emissions. The company that commissioned the study provided data on GHG emissions of all feed types used on the farms within the scope of this evaluation. Feed GHG emissions data was produced by Wageningen Livestock Research and Blonk Consultants. These partners also developed the FeedPrint (http://webapplicaties.wur.nl/software/feedprintNL/index.asp accessed on 16 May 2022) tool, which quantifies GHG emissions in animal feed production and utilisation. The scope of the GHG emissions in the feed production assessment included crop production, feed production, storage, and transport between all phases of production. The GHG emissions (excluding biogenic carbon) of the feeds include land use (LU) and direct land use change (dLUC). Further information about the methodology used in the FeedPrint project can be found in [46]. The GHG emissions of the feeds used in this study were in the range of 445–517 g CO_2_ eq./kg feed.

#### 3.2.2. Breeder Farm and Hatchery

Primary data for the breeder farms (rearing and breeding) and hatchery was collected from six rearing farms, twelve breeding farms, and one hatchery; these data represented all existing breeder farms and hatcheries operated by the company. The scope of the breeder farm evaluation covered the parent farm, including the transport of the parent chicks to the farm. The impact from the great-grandparent and grandparent farms was excluded from the analysis because a previous study found that such impacts were not significant (<1%) [8]. Table 5 shows the main inventory data for the breeder farm and hatchery per FU. SANDACH waste—byproducts of animal origin, not intended for human consumption—was considered rendering material. In this study, waste considered as SANDACH mainly came from the hatchery and included defective (unfertilised) eggs, shells, dead and unviable chicks, and other organic remains.

#### 3.2.3. Grow-Out Farm and Slaughterhouse

Data for the grow-out farms was collected across 30 representative broiler farms out of a total of 155. The inventory for the remaining farms (125) was extrapolated according to the farm size. Data for the slaughterhouse was obtained from one slaughterhouse, which was the only one operated by the company. Grow-out farm performance data for the period of the study is shown in Table 6, including the feed conversion ratio (FCR),. FCR is the “measure of the efficiency with which an animal converts feed into tissue, usually expressed in terms of kg of feed per kg of output (e.g., live weight or protein)” [18]. Table 7 shows the inventory data per FU for the grow-out farms and slaughterhouse. Unlike the other rendering materials from the farms (breeder and grow-out) and hatchery, which were treated as waste, the inedible byproducts from the slaughterhouse have economic value, so they were treated as a co-product and allocated environmental burdens based on their economic value (see Figure 2).

## 4. Results, Discussion, and Sensitivity Analysis

### 4.1. Results and Discussion: Whole Carcass, without Packaging (P1)

**Figure 3:**
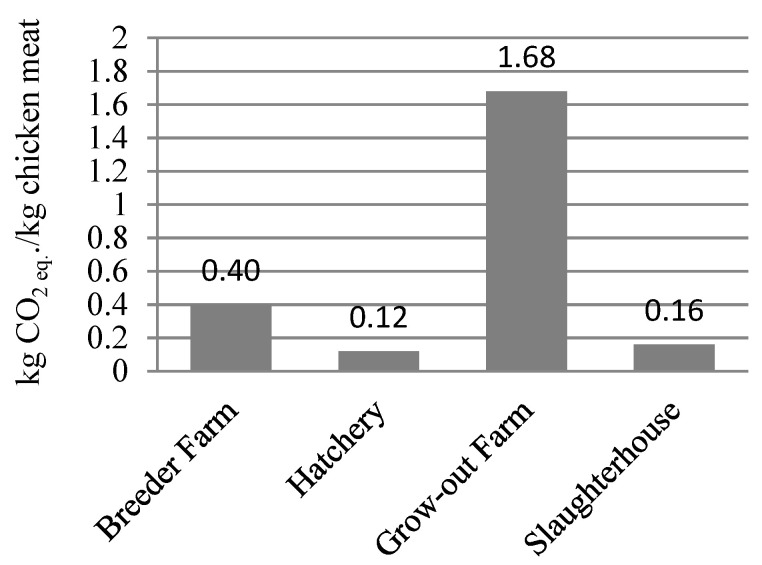
GHG emissions from the life cycle stages evaluated within the chicken meat supply chain.

**Figure 4:**
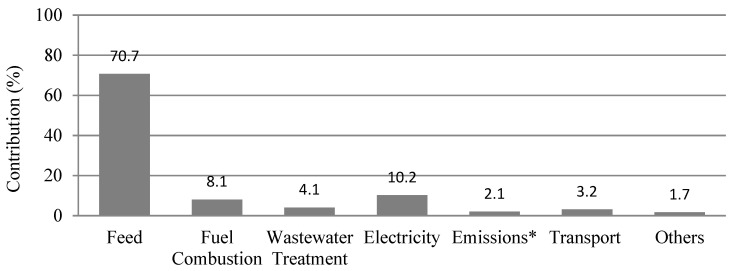
Contribution to GHG emissions from major sources within the processes evaluated in chicken meat production. * Emissions from enteric fermentation and on-farm manure management.

The results (see Figure 3) show that the climate change (CC) impact per kg of whole carcass (P1: without packaging) was 2.36 kg CO_2_ eq. This result is in line with results from the literature discussed in Section 2 (mostly in the range of 2.46–3.30 kg CO_2_ eq./kg carcass). Figure 3 shows that the grow-out farm was the largest contributor (1.68 kg CO_2_ eq.) to the GHG emissions, due to its large consumption of feed. This was followed by the breeder farm (0.40 kg CO_2_ eq.). Within input/output sources (see Figure 4), the major contributors to CC were feed production (70.7%), followed by electricity use (10.2%), and fuel combustion (8.0%). In Europe, most of the soy used in the feed is imported from the United States of America, Argentina, or Brazil [23,47]. Soy from Argentina and Brazil contributes largely to the feed’s CC impact due to land use change [7,9]. The main recommendation here is for feed production companies to produce feed with the lowest impact possible (taking into account composition, origin of ingredients, etc.). Another way the CC impact can be reduced may be for poultry farmers to improve the feed conversion ratio (FCR), thus reducing the amount of feed needed for a certain amount of meat production. This could lead to reductions in the CC impact of the feed, both at the feed production level and on the broiler farm [8]. At the farm level, the FCR could probably be improved by ensuring proper health status (via handling, vaccination, disease control, house disinfection, etc.) of the birds, and through proper farm management practices, such as water management, feed management, temperature control, adequate ventilation, lighting, among others [48]. However, in cases where the FCR is close to its biological minimum, such as in the case of this study (1.7), the focus should be on reducing the impacts of feed production (composition, origin of ingredients, etc.).

Apart from feed production, other major contributors to the CC impact were fuel combustion for heat generation on the farms (8.0%), electricity use on the farms and in the slaughterhouse (10.2%), and wastewater treatment (4.1%). Electricity use in the slaughterhouse was almost half (48.1%) of the total electricity use on the farms. Therefore, investing in energy efficiency and clean energy and electricity production systems (such as electricity generation from chicken litter/manure) could be explored to reduce GHG emissions in the chicken meat supply chain [49].

### 4.2. Results and Discussion: Meat Cuts (P3, P4, P5, and byP6) and Packaging

Production of meat cuts requires an additional electricity input (0.061 kWh/whole carcass) for the cutting process. The burdens from this process need to be allocated, based on mass or economic value, to each of the products obtained (see Table 4). This allocation also affects the burdens from all upstream processes prior to cutting (feed production, farming, and slaughterhouse).

According to the ISO 14044, allocation needs to be as close as possible to the causality of the burdens. In the present case, byP6 is not responsible for poultry production because, although it represents a significant weight (27% of the whole carcass), it has a low economic and food value (as it mainly consists of bones, cartilage, and flesh), and only 30% of it is sold for human consumption (to make broth). Therefore, byP6 has a lower contribution to the burdens than P3, P4, and P5. This fact needs to be reflected in the allocation method chosen.

**Figure 5:**
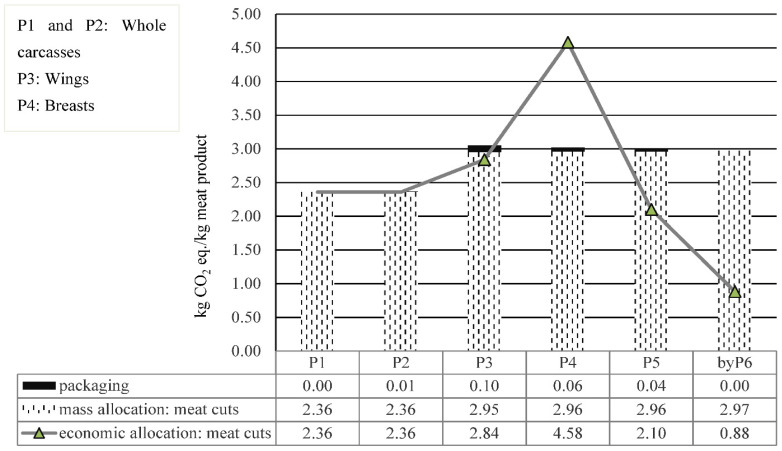
Influence of packaging on the results of four products and the impact of the choice of allocation on the meat cuts.

Results of CC impact per kg of each meat cut, together with the impact of packaging for each of the products studied (P1, P2, P3, P4, P5 and byP6), are shown in Figure 5. The packaging had a relatively small contribution to CC impact for all products studied, when considering the chicken life cycle from cradle-to-slaughterhouse; 0.4% for the whole carcass (P2) and 1.3–3.4% for the meat cuts (P3, P4 and P5).

When mass allocation was applied to the meat cuts (Figure 5), there was no significant differences in the CC results (2.96 kg CO_2_ eq./kg meat) among the different meat cuts, including byP6, even though byP6 is a byproduct with lower economic value (see Table 4). The higher impact per kg of meat cuts compared with the whole chicken carcass (P1 and P2) was due to meat loss during cutting, consisting of the 70% of byP6 not used for human consumption, which is treated as a residual and therefore has no impact. On the other hand, applying economic allocation to the meat cuts showed differences in the CC results, with byP6 having the lowest impact (0.88 kg CO_2_ eq./kg) (see Figure 5), which makes more sense. This difference was due to the real meat content in the different cuts, resulting in price variations (i.e., byP6 is the one containing less meat, as it is mainly bones). Therefore, economic allocation proved to be a better allocation method for the meat cuts (more related to the causality of the process). This allocation method has also been applied in similar studies [8,26]. Nonetheless, one of the drawbacks of economic allocation is the price volatility of products over a set period [26]. For this reason, a sensitivity analysis was performed in the next section (Section 4.3.1) using price changes of the meat cuts over a 10-year period.

One of the novel contributions of this paper was the analysis of the differences in the CC results between the whole carcass and meat cuts (Figure 5). The results showed that the impact of meat cuts was significantly higher than that of the whole carcass, although the additional energy required by the cutting process contributed very little to this increase (Increase in GHG emissions due to cutting was only ~1.3% of the total). In view of this, it is important to distinguish between the whole carcass and meat cuts, especially when benchmarking results against similar studies. In this regard, previous studies found similar results. For example, Wiedemann et al. [8] found that boneless chicken portions (cuts) had 39% more CC impact than the whole carcass, whereas Bengtsson et al. [26] observed that breast fillet had three times the CC impact of whole roast chicken. Nevertheless, these studies did not provide detailed analyses or explanations of the results.

Despite the differing impacts observed according to the type of product analysed in this study, the LEAP guideline does not make any distinction in terms of allocation between chicken meat cuts and the whole carcass. It recommends that these products be treated as equivalent and therefore no allocation is given to the different meat cuts. The drawback of this recommendation is that this is an important difference (due to economic and food value of the different cuts and the mass loss resulting in edible offal) that should be considered. Moreover, there is an increasing need for companies to know the environmental impact of each of their marketable products [8,26,34]. This trait is not specific to chicken meat, but also important for other meat products, such as beef, where there is a difference in the climate change impacts of different beef products [50].

### 4.3. Sensitivity Analysis

#### 4.3.1. Allocation to Meat Cuts

**Figure 6:**
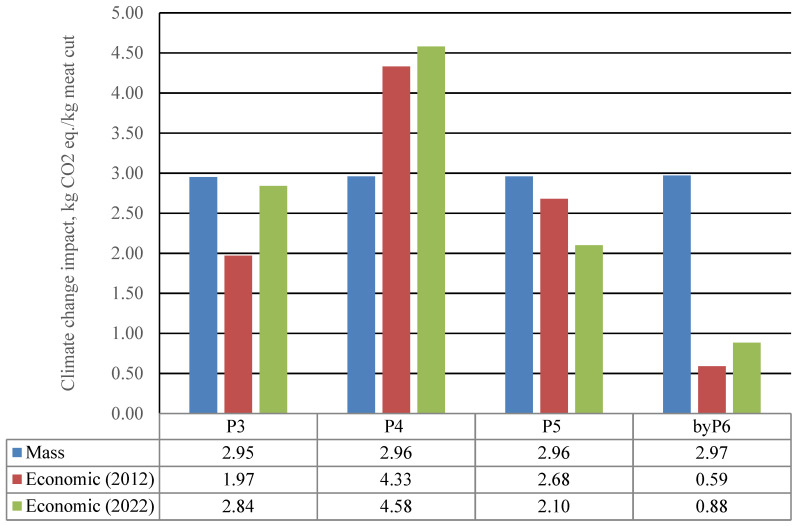
Impact of different allocation choices on CC results of the meat cuts.

The LEAP guideline [18] recommends that sensitivity analysis is performed to test the robustness of the choice of an allocation method on the outcome of the study. For this reason, we analysed the robustness of economic allocation over a period of 10 years (2012–2022). To avoid price variability, it is recommended to use the average price of a product over a 5-year period [46]. However, in the Spanish market, the only data available was average price per kg whole carcass, which increased about 46% during the period 2017–2022 [51], Thus, in the present study, price data from the studied company were used (see Table 8), for each of the chicken products assessed. Climate change impact results show a considerable difference in the last 10 years, according to changes in consumer preferences. For instance, though P3 (wings) had appreciated from 10 years ago, P5 (leg quarters) had lost favour (see results in Figure 6). These variations due to consumer preferences could be avoided if the protein or food value content per kg was used in the allocation method instead of the price.

#### 4.3.2. Food Waste

**Figure 7:**
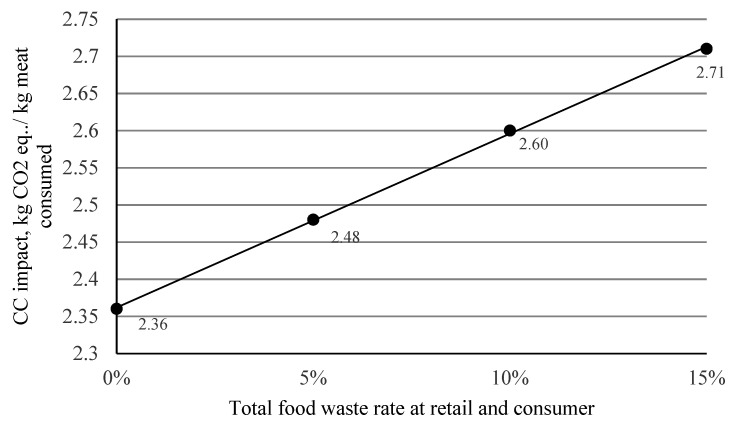
Sensitivity of CC impact to food waste at retail and consumer level.

One of the main problems in food supply chains is the large amount of food waste that occurs at the retail and consumer ends [52,53,54]. In the case of meat products, the total food waste at retail and consumer level is about 15% (4% at retail and 11% at the consumer) [53]. Moreover, for chicken meat, a Flemish study found that the average waste rate at the consumer level, for major wasters in Flanders (a Belgium region), is ~8% slightly less than the average for meat waste in Europe [55]. This quantity of chicken meat is produced but will never be consumed. Thus, if a functional unit (FU) that reflects food waste is taken, such as “1 kg of chicken meat consumed,” the real impact per FU will increase depending on the % of food waste (see Figure 7). Figure 7 shows that reducing the total food waste from 15 to 10% leads to a ~4% reduction in CC impact per kg chicken meat consumed, and this trend continues for any further reductions in the food waste. The more meat that is wasted (at retail and consumer levels), the more meat will have to be produced so that the same amount can be consumed (1 kg), and this is without considering the increased need for waste treatments (with the corresponding increased impact). This indirect impact, due to food waste, is usually not attributable to food production, but rather to packaging and consumer attitudes [56,57]. Education and consumer awareness, together with packaging, can play an important role in preventing this waste at the retail and consumer ends of the food supply chain [57,58].

For meat products, such as chicken meat, one of the ways to reduce food waste is to preserve the meat as long as possible, extending its shelf life. For fresh meat products, packaging with modified atmosphere has been shown to be an effective way to extend its shelf life [59]. Although the production of this packaging may have a slightly higher impact, the overall impact in the meat-packaging supply chain will probably decrease, due to food waste reduction [58]. Thus, research on new and sustainable packaging options for chicken meat should be strongly encouraged.

## 5. Conclusions and Future Trends

The climate change impacts of four chicken meat products (whole carcass, wings, breast fillets and leg quarters) from cradle-to-slaughterhouse gate were evaluated in this study. Detailed analyses and discussion of mass and economic allocation of burdens to the meat cuts suggested that economic allocation is preferable as it better reflects the causality of the process. In addition, the results showed that a distinction should be made between the whole carcass and meat cuts, especially when benchmarking results against similar studies, because there is a significant increase in climate change impact per kg of meat cut compared with the impact per kg of the whole carcass. This is due to the mass loss in the form of an edible byproduct (mainly consisting of bones and cartilages) scarcely used for human consumption (30%). This result is especially important because the LEAP guideline recommends that the whole carcass and meat cuts are treated as equivalent. A sensitivity analysis using price changes over a 10-year period (2012–2022) revealed that impact results could change according to changes in consumer preferences over time; thus, setting an average price over a period (for example, 5 years), or even an allocation based on food value content instead of price, could improve the reliability of the results.

This paper also provided, for the first time, disaggregated inventory data for the chicken meat production chain in Spain; this includes data from breeder farms, hatcheries, grow-out farms, and a slaughterhouse.

In addition, the study showed that chicken farms contributed the most (88.1%) to climate change impact per kg of whole carcass, and this was largely due to the impact of feed production (70.7% of total). Some options to reduce feed impact could be sourcing feed with lower impacts, by considering feed origin and composition, and improving the feed conversion ratio (FCR) at the farm level whenever possible. Fuel combustion for heat generation on the farms and electricity-use across the meat production chain also largely contributed (18.2%) to the climate change impact. Therefore, increasing energy efficiency and renewable energy use should be further explored.

Packaging production did not significantly contribute to the climate change impacts of the chicken products when considering all evaluated life cycle stages (cradle-to-slaughterhouse gate). The contribution of the packaging was 0.4% of the climate change impact for the whole carcass and 1.3–3.4% for the meat cuts from cradle-to-slaughterhouse gate.

Finally, it is worth mentioning that future initiatives to reduce the impact of chicken meat production should focus on developing lower impact diets for broilers, such as low protein diets supplemented by amino acids [32] and on sustainable meat packaging systems to prevent food waste across the chicken meat supply chain (for instance, reducing food waste at the retailer and consumer level by 5% could decrease the climate change impact due to associated chicken meat production by 4%).

## Figures and Tables

**Table 3:** Main chicken meat products studied and their packaging types.

Product ID	Chicken Meat Product	Packaging Type
P1	Whole carcass	No packaging
P2	Whole carcass	PE bag
P3	Chicken wings	PET tray with PET film
P4	Chicken breasts	PP-PA bag
P5	Chicken leg quarters	PS-PVC tray with PVC film

PE: Polyethylene; PET: Polyethylene terephthalate; PP: Polypropylene; PA: Polyamide; PS: Polystyrene; PVC: Polyvinyl chloride.

**Table 4:** Allocation criteria for chicken meat cuts.

Product	Mass (kg/Whole Carcass)	€/kg (Year: 2022)	Allocation	
			Mass (%)	Economic (%)
P3 (wings)	0.21	4.42	13.5	13.0
P4 (breasts)	0.54	6.95	35.5	55.0
P5 (leg quarters)	0.62	3.25	40.9	29.0
byP6 (edible offal)	0.51 *	1.24 *	10.1	3.0

* Total mass of the whole carcass was 1.88 kg. For the edible offal, only 30% is sold (1.24 €/kg) as human food; the rest (70%) is used as animal feed, with no substantial revenue accruing from it. Hence, it was treated as a residual in this study. Therefore, the mass and economic allocation values are based on 30% of the edible offal.

**Table 5:** Main inventory data for breeder farms and hatchery per FU.

Life Cycle Inventory	Values	
Inputs	Breeder Farm	Hatchery
Day-old chicks (Parents)	5.17 × 10^−3^	-
Feed, kg	2.56 × 10^−1^	-
Electricity, kWh	1.34 × 10^−2^	9.59 × 10^−2^
Propane, kg	8.93 × 10^−4^	7.61 × 10^−4^
Diesel, kg	1.17 × 10^−3^	1.01 × 10^−4^
Water, kg	6.45 × 10^−1^	3.47 × 10^−1^
Bedding (wood shaving/rice husk), kg	3.82 × 10^−3^	-
Cleaning and disinfection, kg	2.08 × 10^−3^	2.34 × 10^−3^
Transport, tonne-kilometre (tkm)	6.58 × 10^−2^	2.96 × 10^−2^
Outputs		
Day-old broiler chick (~40 g)	-	5.61 × 10^−1^
Wastes:		
Wastewater, m^3^	6.50 × 10^−4^	2.92 × 10^−4^
Rendering material, kg	2.18 × 10^−3^	-
SANDACH *, kg	-	3.20 × 10^−2^
Sewage sludge, kg	-	5.41 × 10^−3^
Emissions (manure and enteric fermentation):		
Methane (CH_4_), kg	1.35 × 10^−5^	-
Nitrous oxide (N_2_O), kg	3.22 × 10^−6^	-

* Byproducts of animal origin, not intended for human consumption. -: not applicable

**Table 6:** Broiler performance data.

Name	Value
Yearly production, birds	24,000,000
Initial weight, g	40
Final weight, g	2590
Average days to maturity, days	44.5
Mortality (%)	5.47
Feed conversion ratio (FCR)	1.70 *

* Calculated using yearly bird production (minus mortality).

**Table 7:** Main inventory for broiler grow-out farm and slaughterhouse per FU.

Life Cycle Inventory	Values	
Inputs	Grow-Out Farm	Slaughterhouse
Broiler chicks	5.61 × 10^−1^	-
Broilers	-	5.30 × 10^−1^
Feed, kg	3.04 × 10	-
Electricity, kWh	9.12 × 10^−2^	1.94 × 10^−1^
Natural gas, kg	-	1.10 × 10^−2^
Propane, kg	2.44 × 10^−2^	-
Diesel, kg	1.94 × 10^−3^	3.42 × 10^−4^
Biomass, kg	3.88 × 10^−2^	-
Bedding (wood shaving/straw/rice husk), kg	1.22 × 10^−1^	-
Water, kg	6.14 × 10	5.66 × 10
Refrigerant (ammonia: NH_3_), kg	-	2.79 × 10^−5^
Cleaning and disinfection, kg	1.59 × 10^−2^	1.97 × 10^−3^
Transport, tonne-kilometre (tkm)	8.48 × 10^−1^	1.73 × 10^−2^
Outputs		
Chicken meat (whole carcass), kg	-	1.00 × 10
Inedible by-products*, kg	-	3.76 × 10^−1^
Wastes:		
Rendering material, kg	7.95 × 10^−2^	-
Wastewater, m^3^	1.75 × 10^−3^	5.56 × 10^−3^
Sewage sludge, kg	-	4.38 × 10^−2^
Emissions (manure and enteric fermentation):		
Methane (CH_4_), kg	9.13 × 10^−5^	-
Nitrous oxide (N_2_O), kg	1.54 × 10^−4^	-

* Inedible byproducts: viscera, feet, blood, feathers, bones, etc. -: not applicable

**Table 8:** Mass and economic allocation criteria for the meat cuts.

	Allocation Type				
	Mass	Economic			
		2012		2022	
Product ID	Allocation (%)	Average Price per kg (€/kg)	Allocation (%)	Average Price per kg (€/kg)	Allocation (%)
P3	13.5	1.97	9.0	4.42	13.0
P4	35.5	4.40	52.0	6.95	55.0
P5	40.9	2.71	37.0	3.25	29.0
byP6	10.1	0.52	2.0	1.24	3.0

P3: wings; P4: breasts; P5: leg quarters; byP6: edible offal.

## Data Availability

Data is contained within the article.
